# A Personalized Intervention to Increase Environmental Health Literacy and Readiness to Change in a Northern Nevada Population: Effects of Environmental Chemical Exposure Report-Back

**DOI:** 10.3390/ijerph21070905

**Published:** 2024-07-11

**Authors:** Johanna R. Rochester, Carol F. Kwiatkowski, Iva Neveux, Shaun Dabe, Katherine M. Hatcher, Michael Kupec Lathrop, Eric J. Daza, Brenda Eskenazi, Joseph J. Grzymski, Jenna Hua

**Affiliations:** 1 Million Marker Wellness, Inc., Berkeley, CA 94704, USA; jo@millionmarker.com (J.R.R.); carol@millionmarker.com (C.F.K.); katherinehatcher@gmail.com (K.M.H.); mike@millionmarker.com (M.K.L.); ericjdaza@gmail.com (E.J.D.); eskenazi@berkeley.edu (B.E.); 2 Healthy Nevada Project, Renown Health, Reno, NV 89557, USA; ineveux@med.unr.edu (I.N.); dabe316@gmail.com (S.D.); jgrzymski@med.unr.edu (J.J.G.); 3 Department of Internal Medicine, University of Nevada, Reno, NV 89557, USA

**Keywords:** endocrine-disrupting chemicals, bisphenols, phthalates, parabens, environmental health literacy, exposure intervention

## Abstract

Background: Interventions are needed to help people reduce exposure to harmful chemicals from everyday products and lifestyle habits. Report-back of individual exposures is a potential pathway to increasing environmental health literacy (EHL) and readiness to reduce exposures. Objectives: Our objective was to determine if report-back of endocrine-disrupting chemicals (EDCs) can reduce EDC exposure, increase EHL, and increase readiness to change (i.e., to implement EDC exposure-reduction behaviors). Methods: Participants in the Healthy Nevada Project completed EHL and readiness-to-change surveys before (*n* = 424) and after (*n* = 174) a report-back intervention. Participants used mail-in kits to measure urinary biomarkers of EDCs. The report-back of results included urinary levels, information about health effects, sources of exposure, and personalized recommendations to reduce exposure. Results: EHL was generally very high at baseline, especially for questions related to the general pollution. For questions related to chemical exposures, responses varied across several demographics. Statistically reliable improvements in EHL responses were seen after report-back. For readiness to change, 72% were already or planning to change their behaviors. Post-intervention, women increased their readiness (*p* = 0.053), while men decreased (*p* = 0.007). When asked what challenges they faced in reducing exposure, 79% cited not knowing what to do. This dropped to 35% after report-back. Participants with higher propylparaben were younger (*p* = 0.03) and women and participants who rated themselves in better health had higher levels of some phthalates (*p* = 0.02–0.003 and *p* = 0.001–0.003, respectively). After report-back, monobutyl phthalate decreased among the 48 participants who had valid urine tests before and after the intervention (*p* < 0.001). Conclusions: The report-back intervention was successful as evidenced by increased EHL behaviors, increased readiness to change among women, and a decrease in monobutyl phthalate. An EHL questionnaire more sensitive to chemical exposures would help differentiate high and low literacy. Future research will focus on understanding why men decreased their readiness to change and how the intervention can be improved for all participants.

## 1. Introduction

Environmental factors (i.e., the exposome) are responsible for more than 70% of the risk of developing chronic diseases [1], especially when exposed before birth [2]. Exposure to environmental chemicals is an important component of the exposome. Bisphenols (such as bisphenol A, BPA), phthalates, and parabens are used in plastics and personal care products, and are endocrine-disrupting chemicals (EDCs) [3,4,5,6]. These chemicals are linked to increases in chronic diseases including type 2 diabetes [7,8], heart disease [5,9], obesity [10,11,12,13,14], and immune disorders [5,15,16]. Of particular concern are the harmful effects these chemicals can have on fertility. EDCs have been linked to infertility in both women and men [5,17], increased miscarriage risk [18], and premature birth [19,20]. EDC exposure has also been shown to reduce in vitro fertilization success for exposures in both parents [5], likely due to effects such as lower embryo quality, reduced implantation success, and a decreased live birth rate [17].

Data from the Centers for Disease Control’s National Health and Nutrition Examination Survey (NHANES) have shown that more than 90% of US adults have detectable levels of common EDCs, such as bisphenol A (BPA) and phthalates, in their urine [21,22]. And, because these chemicals have very short half-lives (6–12 h) [23,24], this high detection level in almost all individuals tested indicates ubiquitous, daily exposure. However, because of the short half-lives of these chemicals, it has been shown that reducing sources of EDC exposures can reduce or eliminate internal EDC exposure biomarkers, in a short amount of time (3 days to 3 weeks) [25,26]. Thus, increased awareness of one’s personal exposome is a crucial component of health optimization.

Environmental health literacy (EHL) is the understanding and knowledge surrounding how certain environmental exposures can impact health. Increasing EHL in the general public is a crucial step in reducing EDC exposures and their adverse effects [27]. The actual level of EHL in the general public in the US is not known, as efforts to quantify it have only just been initiated within the last few decades [27]. However, most experts agree that efforts are needed to increase EHL in the general public [27,28,29], especially in low-income communities, marginalized communities, and communities of color, all of which are at greater risk of toxic exposures due to societal inequities [28,30,31,32].

Furthermore, there is a known gap between EHL and the ability to display pro-environmental behavior [33]. Thus, increasing EHL is not enough. Rather, increasing EHL must also lead to behavior changes in order to reduce exposures. In this study, we explore actionable report-back as a method to increase both EHL and exposure-reduction behaviors. “Report-back” is defined as “the process by which personalized exposure findings are shared with the participant” [34,35]. Biomonitoring studies have found that effective report-back of internal exposures increased motivation for personal EHL education and had the potential to promote community engagement and effective science translation [36,37,38,39,40,41,42]. However, there has been less research examining the effectiveness of personal internal and external exposure report-back on increasing EHL and motivation for daily behavior changes.

With the expansion of direct-to-consumer testing related to genetics, COVID-19, and an expansion in tele-health [43,44,45], it is clear that individuals can be motivated to increase health literacy and healthy behaviors from personalized testing and report-back [46,47]. Understanding personal exposure levels and daily sources of EDC exposures are instrumental in increasing EHL. However, there are few tools or resources for individuals to learn and understand their EDC exposures and proactively assess, track, and reduce them. Million Marker (MM), a recently established biotech company, has developed a direct-to-consumer urine test to track internal exposures to several EDCs (i.e., bisphenols, phthalates, parabens, and oxybenzone). This information, along with personal exposure surveys, allows MM to provide report-back of EDC exposures and personalized exposure reduction recommendations to customers [48]. However, the ability of the MM test and report-back service to increase EHL and/or motivate exposure-reduction behavior changes has not been previously validated.

Recently, the National Institute of Environmental Health Sciences (NIEHS) highlighted several gaps in the understanding of EHL in the population, such as whether there are specific aspects of EHL amenable to intervention, whether increased EHL improves health outcomes, and whether risk messaging about environmental factors leads to behavior changes [27]. Thus, NIEHS has called for increased research into EHL, with particular focus on community engagement, including topics such as (1) processes for increasing environmental health literacy, (2) developing and validating measures of EHL at both individual and community levels, (3) assessing the effectiveness of existing environmental risk messages, and (4) measuring the extent of behavior change based on health-risk messaging [27].

MM has partnered with the Healthy Nevada Project (HNP) to begin to address these EHL research needs. The HNP is a population health and genetics research study, with health and genomic data collected from more than 50,000 participants [49]. The study has also been returning clinical results to study participants since late 2018. Participants in the HNP provide informed consent, are asked to fill out personal health surveys, and provide samples for genomic testing [50]. The HNP partners with private companies (i.e., academic–industry partnership model) to enable cost-effective, scalable, and innovative research [51,52]. The MM-HNP collaboration allows for expansion into exposomic study of this population, as well as future research into gene–environment interactions.

In this study, we aimed to (1) assess the baseline EHL, readiness to change (i.e., reported willingness to reduce exposures through lifestyle changes), and internal EDC levels of reproductive-aged men and women in the HNP population, (2) compare changes in these variables before and after a non-randomized report-back intervention, (3) assess the relationship of these variables with several demographics (age, sex, race/ethnicity, education, income, BMI, and health status), and (4) determine the usability of the MM service (i.e., system usability score, SUS).

## 2. Methods

All study methods were reviewed and approved by the University of Nevada, Reno, General Institutional Review Board (IRB), which oversees social, behavioral, and educational research and biomedical research and clinical trials (IRB00000215; “[1786153-1] Renown Institute for Health Innovation-Million Marker Detect and Detox Pilot”, approved 19 July 2021). Participants were not compensated for their participation.

### 2.1. Population

**Figure 1:**
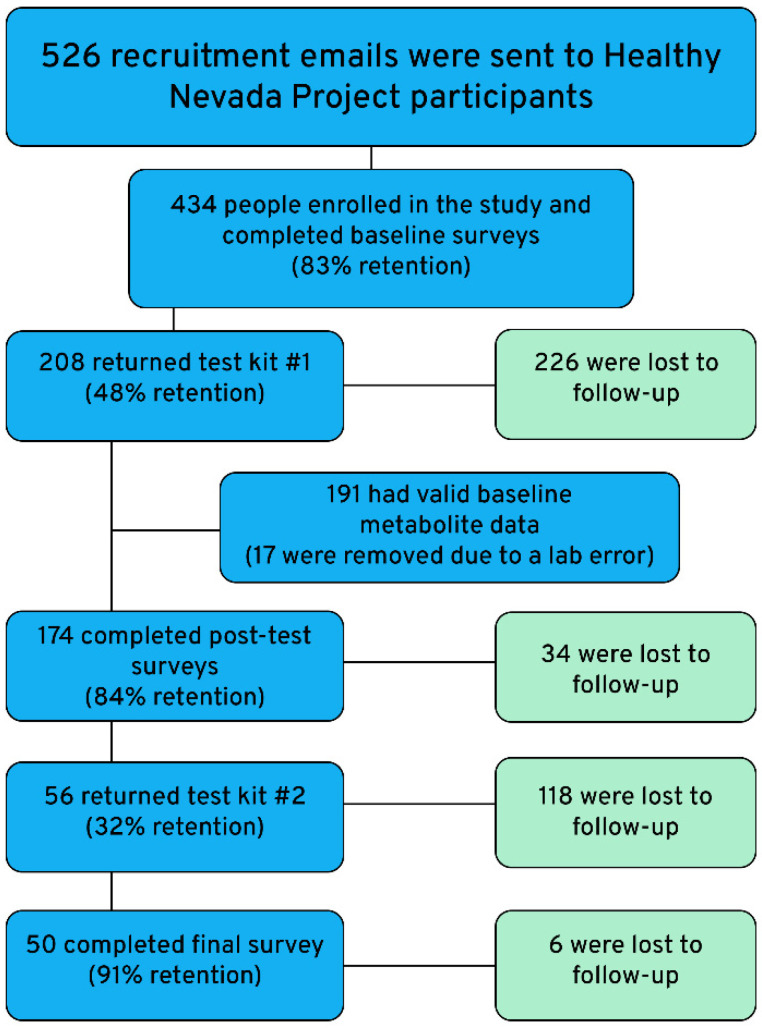
Participation flow chart.

Recruitment, consent, and data collection to the HNP are described in detail in Grzymski et al. [50]. Between August 2021 and July 2022, 526 recruitment emails describing the study and asking for participation were sent to HNP participants. Eligible participants were aged 18–40, had a smartphone, spoke English, were not pregnant, and had no known diagnoses for cancer, metabolic disease, or kidney disease. As shown in Figure 1, from these emails, 434 individuals agreed to participate in the study and were sent online consent forms and surveys, as well as MM testing kits. We received complete pre-test surveys and biomarker data from 208 individuals and pre-test surveys, post-test surveys, and biomarker data from 174 individuals. Of these, 56 submitted a second urine sample for biomarker analysis and 50 people completed a brief closing survey.

### 2.2. Demographic Information and Study Surveys

Participants’ age, sex, race/ethnicity, level of education, income, height, weight, and self-assessed health status were collected via questionnaire during recruitment to HNP. Once recruited to the present study, participants completed additional surveys including EHL, readiness to change, and digital literacy. The digital literacy survey was adapted from The Digital Health Literacy Instrument [53] and consisted of 3 questions with the aim of determining participants comfort level with online tools and mobile phone applications (assessed at baseline only).

The EHL survey was adapted from the “General Environmental Health Scale” [29]. In the original survey, subscales were created to assess EHL knowledge, attitudes, and behaviors and the results were analyzed within each scale. Because we modified the questions to make the survey more specific to chemical exposure EHL, the subscale analysis would have sacrificed sensitivity; instead, we analyzed the relevant questions individually. The readiness to change survey was developed using the transtheoretical model of health behavior change [54] and included questions that reflect behaviors related to the reduction in EDC exposure. The System Usability Scale (SUS) was adapted from Brooke and included in the post-intervention survey [55]. The SUS is the survey used by the U.S. Department of Health and Human Services to measure the usability of products and services, including hardware, software, mobile devices, websites, and applications [56]. All survey questions underwent review by assessors with in-depth knowledge of the HNP population, and questions were tailored to the specific needs and attitudes of this population.

### 2.3. Self-Report Exposure Data

Exposure data were collected via a journaling tool available on the mobile application platform [48]. Participants were asked to fill out a 24 h exposure journal to log potential sources of exposure, such as from personal care products, food and drinks, and other household products used and lifestyle activities within the past 24 h before urine sample collection. Questions were both multiple choice and open-ended. Completion of the journal was not a requirement but aided in personalization of the report-back, as described below. Data from exposure journals were not analyzed for this study.

### 2.4. Biomarker Measurement

After pre-test survey completion, participants were mailed a MM Detect & Detox Kit (Million Marker, Berkeley, CA USA) [48], consisting of a polypropylene urine sample collection cup, return packaging, return label, and directions for sample collection. Users were also directed to detailed sample collection instructional videos on the MM website. These directions/videos are designed to minimize any contamination during sample collection. Kits were registered to participants via the MM app, available to download for Android or iOS. Samples were then collected from participants’ first void in the morning, the day after the (optional) 24 h exposure journal. Participants were instructed to not freeze samples. Participants mailed back the samples to a third-party analytical laboratory via 2-day shipping. A previous study was conducted to demonstrate the stability of urinary metabolites during shipping and found no degradation of metabolites over several days at room temperature and several freeze/thaw cycles. FedEx Priority Overnight for shipping was used to ensure the sample was delivered to the lab as quickly as possible. Samples were logged upon receiving and immediately aliquoted and stored in a −80 °C freezer. The urine metabolites analyzed were bisphenol A (BPA), bisphenol S (BPS), bisphenol F (BPF), monobutyl phthalate (MBP), monoethyl phthalate (MEP), mono(EthylHexyl) phthalate (MEHP), mono-(2-ethyl-5-hydroxyhexyl) phthalate (MEHHP), mono-(2-ethyl-5-carboxypentyl) phthalate (MECPP), methylparaben (MePB), ethylparaben (EPB), propylparaben (PPB), butylparaben (BUP), and oxybenzone (OBZ). These are major urinary metabolites of BPA, BPA alternatives, phthalates, parabens, and oxybenzone found in more than 95% of the US population [21,57,58,59,60].

The urine samples were analyzed via liquid chromatography/tandem mass spectrometry (LC/MS/MS) following sample preparation. In brief, 100 μL of urine was added to 100 μL of water, isotopically labeled standards were spiked into samples and blank water, and cocktail standards were spiked at 5 μL each. Samples and standards were incubated with 25 μL β-Glucuronidase buffer for 2 h at 37 °C, and 275 μL of water was added to vials. Solvent blanks were prepared concurrently. Samples were injected in duplicate, and blanks were inserted after every duplicate sample. Agilent Prochell 120-EC18 column (Agilent Technologies, Santa Clara, CA USA) was used.

We minimized batch effects through several steps, including (1) freezing samples until analysis; (2) analyzing all samples in a single batch for each LCMS setup to eliminate batch variation; (3) analyzing a standard mixture of known benchmark metabolites within each batch at multiple time points to control for fluctuation in instrument performance; and (4) using technical blank samples periodically throughout sample collection to control for variation in sampling. To overcome identification inefficiency, we used post-acquisition strategies such as running available standards and representative samples to identify unannotated metabolites that were differentially expressed over time with high confidence. The resulting elution time and mass fragmentation pattern were used as evidence of the potential chemical identity of unannotated metabolites.

### 2.5. Non-Randomized Report-Back Intervention

MM reports were provided to participants via the MM mobile app or PDF. Information was presented in easily understandable graphics and written at an 8th-grade reading level. Participants were given their internal exposure levels of BPA, BPS, BPF, the five phthalate metabolites (which were combined as low molecular weight (MBP and MEP) and high molecular weight (MEHP, MEHHP, and MECPP) metabolites), MePB, EPB, PPB, BUP, and OBZ. Additionally, users were presented with their levels compared to NHANES levels, as well as other MM users’ levels (including MM customers and HNP participants), reported as percentiles (0 to <25: low; ≥25 to <75: medium; ≥75 to <95: high; and ≥95: very high).

For each chemical, participants were provided information about health effects, sources of exposure, and personalized recommendations to reduce exposures based on their biomarker levels and their exposure journal information (if available). Additionally, participants who filled out their exposure journal received detailed product audit results, with problematic ingredients identified and flagged; personalized suggestions for product swaps to lower-toxic products; and/or behavior modification recommendations (i.e., “Reduce consumption of canned foods”, “Eat more home cooked meals rather than eating out”, and “Limit touching receipt paper”). An example of the report is present on the Million Marker website [61].

While waiting for their reports, participants were kept engaged by weekly emails, updating them of the status of their sample and report, as well as providing links to information about EDCs and exposure reduction, including online blogs, toxic-free guides, fact sheets, and lists of low-toxic products [62,63].

### 2.6. Data Analysis

A priori hypotheses were that (1) at the baseline assessment, EHL, readiness to change, and biomarker concentrations would vary by demographics; (2) post-test, EHL and readiness to change would increase, and biomarkers would decrease; and (3) usability of the MM service would be rated highly. For all analyses, data were reviewed to ensure assumptions were not violated [64]. In some instances, variables with response categories with too few participants were combined to avoid violating assumptions of statistical tests. Also, when outcomes were not sufficiently normally distributed for *t*-tests to be valid, nonparametric tests were conducted to compare ordered ranks—not means—across categories. Attrition analyses were run to compare demographic variables between participants who completed post-test surveys and those who did not.

### 2.7. Survey Data

Summary data and associations between demographic variables and EHL and readiness to change were analyzed for the 434 participants with pre-test survey data. Digital Health Literacy was also summarized for this sample. Independent sample *t*-tests, one-way ANOVAs, and Spearman’s rank correlations were run to compare EHL questions across each demographic variable (age, sex, race/ethnicity, education, income, BMI, and health status). To assess associations between demographic variables and readiness to change, Chi-square tests and Spearman’s rank correlations were run. Changes from pre-test to post-test in EHL and readiness to change were analyzed for the 174 participants who completed both surveys using Wilcoxon signed rank tests. Changes in the proportions of participants identifying various challenges to changing their behaviors were assessed using McNemar’s exact tests. System usability responses were summarized and reported.

### 2.8. EDC Biomarker Data

Differences in EDC metabolite quartiles by demographic variables were assessed using Mann–Whitney U tests for variables with two categories and Kruskal–Wallis H tests for variables with more than two categories. Changes from pre-test to post-test were assessed using paired samples *t*-tests.

### 2.9. Statistical Significance

A *p*-value was considered statistically significant if it was <0.05. As a general practice throughout the paper, we have used language to avoid the common misconstrual of “statistical significance” as an indicator of whether a finding is clinically or practically meaningful, rather than the correct understanding of “statistical significance” as an indicator of whether the evidence for a true finding (which is only estimated in any given study) is statistically supported or reliable [65]. For example, we use words such as statistically “supported”, “discernible”, or “reliable” when describing findings and reserve “statistically significant” for describing specific *p* values [65,66,67,68,69,70,71,72,73,74]. For convenience and in keeping with common practice, we shorten more scientifically accurate phrases like “statistically reliable sample-based estimate of a true but unknown target-population mean difference” to “statistically reliable difference”.

## 3. Results

### 3.1. Attrition

As shown in Figure 1, recruitment retention was high (83% of those recruited completed baseline surveys); however, only 48% of those who completed baseline surveys returned their urine test kits for analysis. Of the 208 individuals who completed the initial survey and returned their test kit, 174 (84%) also completed post-test surveys, again showing good retention for survey completion. A comparison of baseline demographics between individuals who completed the post-test survey and those who did not revealed no statistically discernible differences. Several survey variables collected through HNP (BMI, education, income, and self-reported health) had missing data for approximately 140 participants on the pre-test surveys and 45 participants on the post-test surveys. Similar to the first test kit, retention was low (32%) for participants returning their second test kit; however, 91% of those who did also completed the final survey. Seventy-eight percent of those who completed the post-test surveys also entered 24 h exposure data into the online app before their first urine test, demonstrating good engagement with the intervention.

### 3.2. Demographics

The demographic characteristics of the 434 individuals who completed the initial survey are reported in Table 1. Participants ranged in age from 18 to 40 with an average age of 31. Approximately 76% were women, 79% were White (the next largest category was Hispanic/Latino with 10%), and over 90% had post high-school education. Income distributions were fairly consistent across the sample, with about 20% in each income category and the majority of participants had at least USD 50,000 in household income. Most considered themselves to be in good or very good health.

### 3.3. Digital Literacy

Digital literacy was extremely high in the study population, with 99.8% of the participants reporting using the internet daily and 91.5% reporting being very comfortable using it. The majority of participants (67.5%) also reported using a fitness or health app weekly to daily.

### 3.4. Environmental Health Literacy

The EHL of the study population was high, particularly for EHL knowledge questions that assessed general environmental pollution from the original survey. Over 99% of participants strongly agreed that secondhand smoking is harmful to health and a cutting board used for raw meat can contaminate other food. Most participants also agreed or strongly agreed with attitudes such as ‘environmental pollution is a problem’ (98%) and ‘there are things I can do to reduce environmental pollution’ (93%). Because these questions were less relevant to the study aims and there was so little variability in responses, they were not analyzed further. The remaining questions are shown in Table 2. In a related question (not part of the EHL survey), 27% of participants said they believed their current level of exposure to chemicals was harming them, only 6% did not believe it, and 66% were unsure.

Statistical tests were conducted on each of the questions in Table 2 to compare baseline values across the demographic variables shown in Table 1. Older participants were more likely to agree with the statement ‘chemicals can be found in carpet, rugs, curtains, and furniture’ (*r_s_*(432) = 0.10, *p* = 0.02). Older participants (*r_s_*(432) = 0.18, *p* < 0.001), those who identified as Hispanic or Latino (*F*(3,429) = 3.16, *p* = 0.02), and those with less education (*F*(4,289) = 5.66, *p* < 0.001) were more likely to agree with the statement ‘chemicals are always bad for my health’. Having a higher BMI was reliably correlated with more frequent (on a scale of never to always) avoidance of inhaling car exhaust (*r_s_*(295) = 0.13, *p* = 0.03). No other demographic differences were statistically reliable.

Wilcoxon signed rank tests were run to compare whether responses to EHL questions went up, down, or stayed the same after the intervention. Post-intervention, more people decreased their agreement with the statement ‘chemicals are always bad for my health, than increased it (*n* = 172; *z* = −2.46, *p* = 0.014), indicating improved EHL. Similarly, more people increased the frequency with which they avoided inhaling car exhaust (*n* = 171; *z* = −3.45, *p* < 0.001) and cleaning products (*n* = 171; *z* = −2.13, *p* = 0.034), compared to the number who decreased these behaviors (indicating improved behaviors), although for all three statements a large number stayed the same.

### 3.5. Readiness to Change

**Figure 2:**
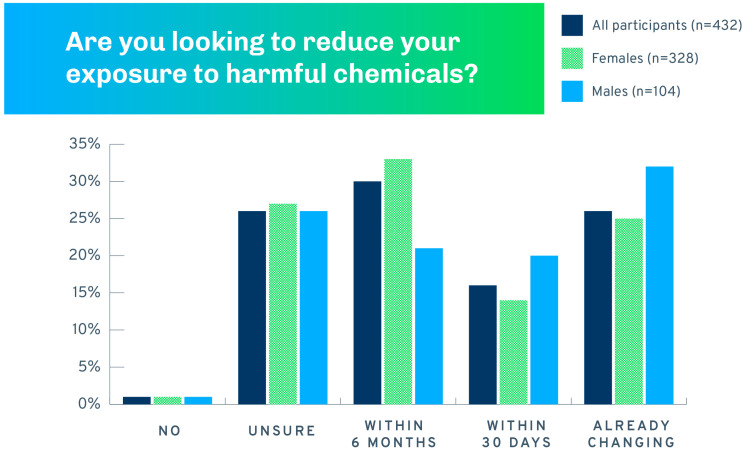
Readiness to change by sex (*n* = 432).

Responses to the question “Are you looking to reduce your exposure to harmful chemicals?” are shown in Figure 2 for the entire pre-test sample. The large majority of participants (72%) were either already changing their behaviors or were planning to do so within the next 30 days or 6 months. Comparisons of readiness to change by the demographic variables in Table 1 indicated that only a difference by sex approached statistical discernibility (χ^2^(3) = 7.02, *p* = 0.071). As shown in Figure 2, more women than men were unsure about reducing exposure or planning to change within the next six months. More men than women were looking to reduce exposure within 30 days or had already changed their behaviors.

**Figure 3:**
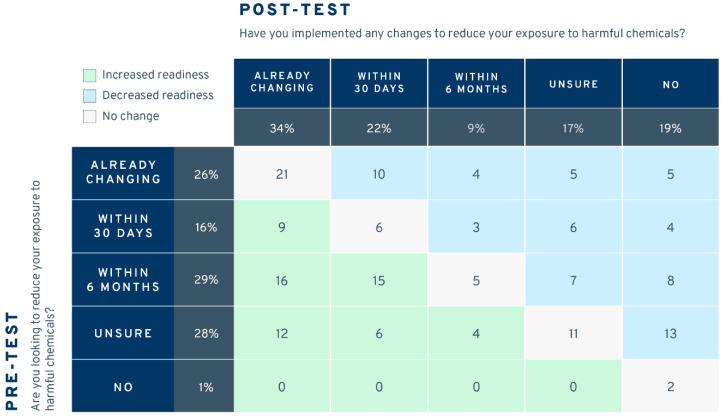
Number of participants responding to pre- and post-test “Readiness to Change” questions (*n* = 172).

**Figure 4:**
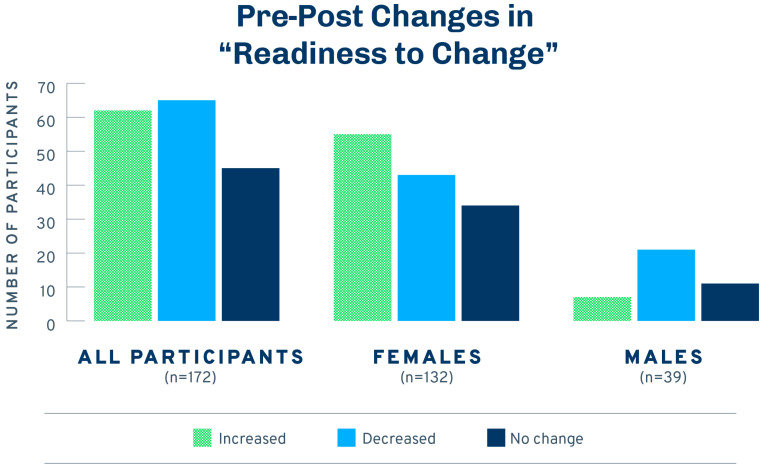
Pre-post changes in “Readiness to Change” for participants who completed post-test surveys (*n* = 172).

Readiness-to-change responses before and after the intervention (among those who participated in both surveys) are shown in Figure 3. The left column shows that 26% of respondents were already making changes to reduce their exposure to harmful chemicals before they received the intervention. A total of 45% were planning to change in the future, 28% were unsure, and only 1% were not ready to make changes. After the intervention (Figure 4, top row), 34% were making changes, 31% were planning to, 17% were unsure, and 19% were not ready or did not think they needed to make changes. A Wilcoxon signed rank test comparing the pre- and post-test median changes indicated no statistically discernible differences in the number of people who increased their readiness to change (*n* = 62), decreased their readiness to change (*n* = 65), or did not change before and after the intervention (*n* = 45; *z* = −0.15, *p* = 0.885; see cell counts in Figure 3).

However, post hoc analyses run separately for women and men revealed interesting differences (Figure 4). Women were more likely to increase their readiness to change than to decrease or remain the same (*z* = −1.9, *p* = 0.053). In contrast, men were more likely to decrease their readiness to change than to increase it or stay the same (*z* = −2.70, *p* = 0.007). To explore this further, we compared the percentages of men and women who believed their current levels of exposure were harming them. Men were more likely than women to say ‘no’ (13% vs. 6%) and less likely to say ‘I don’t know’ (60% vs. 68%; χ^2^(2) = 6.16, *p* = 0.046).

**Figure 5:**
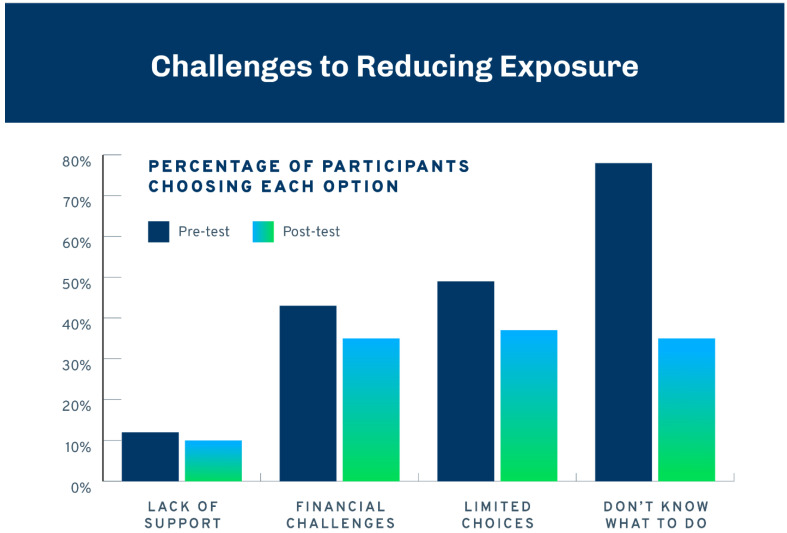
Challenges to reducing exposure to harmful chemicals.

Participants were also asked what challenges they anticipated, faced, or prevented them from making changes to reduce their exposure to harmful chemicals, with the option to select more than one reason. Among the pre-test sample of participants, the most frequent reason cited was not knowing what to do (79%). The next highest responses were having limited choices (46%) and financial challenges (45%). Only 13% cited a lack of support from family and friends. Similar percentages were found among the subset of participants who completed the post-test survey. Changes in this subset before and after the intervention in the challenges they faced are shown in Figure 5. All response categories decreased between the pre- and post-test surveys, with statistically reliable changes (using exact McNemar’s tests) for financial challenges (*p* = 0.04), limited choices (*p* = 0.01), and not knowing what to do (*p* < 0.001).

### 3.6. EDC Biomarker Data

Data from individuals with metabolite concentration values less than the limit of detection (LOD) were replaced by the LOD divided by 2, as suggested for highly skewed data [75]. After applying this transformation to relevant metabolite values, metabolite concentration values for all metabolites were adjusted for specific gravity (SG) to obtain a urinary dilution-corrected concentration for each participant using the following formula: adjusted biomarker concentration = observed biomarker concentration × ((study sample SG Median − 1)/(individual SG − 1)). Of the 208 participants with EDC biomarker data, 17 did not receive specific gravity measurements due to an error at the lab and, thus, were removed from further biomarker analyses. For comparison with NHANES data, which adjusts for creatinine, SG-adjusted values were divided by 1.48 [76,77]. Due to the extreme positive skew of the biomarker data (regardless of transformation), concentration values were divided into quartiles and treated as ordinal data for analysis. Specifically, the ordinal ranks of the values were assessed, rather than the values themselves.

As shown in Table 3, for several biomarkers (MEHP, MEHHP, BPA, BPF, BPS, EPB, BUP, and OBZ), fewer than 25% of participants had concentrations above the LOD. These biomarkers were removed from further analyses. Table 3 also shows that most of the metabolites measured in the HNP population were present at lower concentrations than the 2017–2018 national averages (i.e., NHANES) [78].

Comparisons across quartiles by each of the previously mentioned demographic variables (Table 4) revealed those in the highest quartiles were younger than those in lower quartiles of PPB exposure. For two phthalates, there was a statistically reliable association with the sex of the participants, demonstrating that women had concentrations of MEP and MECPP in the higher quartiles, while men had concentrations in the lower quartiles. There was also a statistically reliable association with the self-reported health rating. Participants who rated themselves as in better health were in the higher quartiles for MBP and MECPP (a similar, nearly discernible association was found for PPB). Analyses of relationships between BMI, race/ethnicity, education, income, and the five metabolites revealed no statistically supported associations.

Post-intervention, urine samples were submitted by fifty-six participants, eight of whom had invalid baseline data and, thus, were not included in this analysis. Concentrations decreased for all but MECPP; however, only MBP was statistically reliable: t(47) = 3.36, *p* < 0.001 (Table 5).

A final brief survey was administered along with the second urine test and 50 people responded. When asked if they made any changes based on the information in their EDC report-back, 26% said no. Fifty percent said they were now using some or all non-toxic personal products, 44% said they were using non-toxic household products, 20% said they dined out less, 32% said they ate less packaged food, 40% used less plastic, and 48% read product labels more.

### 3.7. System Usability Scores

The system usability scores (SUSs) were generally high. Combining responses of ‘agree’ with ‘strongly agree’ and ‘disagree’ with ‘strongly disagree’, most users thought the app was easy to use (60%) and did not think it was unnecessarily complex (51%) or had too many glitches (58%). However, the proportion who thought otherwise (or were undecided) was substantial enough to indicate improvements are needed. Nearly all the participants thought the urine collection and shipping processes were easy (96% and 94%, respectively). They also generally agreed the report was easy to understand (67%) and helpful (58%).

## 4. Discussion

This pilot study demonstrated that intervention via report back of personal environmental exposures, along with personalized recommendations, can result in notable improvements in behavioral and exposure reduction outcomes. Readiness to reduce exposure to harmful chemicals increased among women, knowledge and behaviors reflecting improved EHL increased, and the percentage of participants reporting that they did not know what to do to reduce their exposure decreased substantially. In addition, of those who completed the final survey, many reported making behavioral changes such as reading product labels more and using more non-toxic personal care and household products. Biomarker concentrations also decreased for most EDC metabolites after report-back, with a statistically discernible reduction in the phthalate metabolite MBP.

### 4.1. Readiness to Reduce EDC Exposures Was Increased in Women, but Not Men, after Report-Back

Readiness to reduce exposures, i.e., readiness to change, increased among women after report-back, but, surprisingly, it decreased in men, even though initially men appeared more ready to change than women. Other studies across a variety of behaviors that use a similar readiness-to-change questionnaire (based on the transtheoretical model) have shown sex differences in both directions. In a study of stroke risk behaviors, men were less ready than women to change [79], while a study of smoking behaviors showed women were less ready than men to change [80]. In a study of cannabis use, men and women were shown to have different motivations and success for changing their behaviors [81]. Readiness to change in the context of harmful chemical exposures, as in our study, differs from most previous research, which assesses well-known risky behaviors such as smoking. In our study, prior to receiving their report-back, participants may have been unaware of the behaviors causing increased EDC exposures and the associated health risks. We found that the percentage of men who did not think their current levels were harming them was twice as high as for women. This sentiment could have carried through to the report-back, where men may have found no need to change. It is also possible that men, more than women, did not want to invest the time, felt overwhelmed, or did not feel empowered to make recommended changes, for example, if they are not the primary shoppers in the household. This finding of sex differences is an important first step toward improving our understanding of EDC exposure-reduction behaviors in men and women and we plan to examine it in more detail in future qualitative and quantitative research.

### 4.2. Environmental Health Literacy (EHL) Is Challenging to Assess, and Demographic Differences Exist

The field of EHL is complex, multidisciplinary, and still relatively new [27]. Among the many challenges in the development of valid and reliable assessments of EHL is the multitude of exposures, health outcomes, and populations of interest. We found our EHL survey, adapted from the only validated published survey, was not sensitive enough to show large changes in EDC-specific EHL, especially when analyzed via subscales. Thus, we are developing a more EDC-specific EHL questionnaire for use in future studies. Others are also working to validate EHL surveys—a recent cross-sectional study identified latent constructs in a phthalate-specific EHL survey that predicted health protective behaviors (phthalate avoidance) among women of reproductive age [82]. The study also identified lower phthalate awareness and knowledge among non-White participants.

Our study also found lower EHL knowledge (based on one question) among those who identified as Hispanic or Latino, as well as older participants and those with less education. Minority and marginalized populations are disproportionately exposed to and affected by EDCs, due to differences in product use, demographics, location, occupations, and lifestyle, largely stemming from social inequalities and systemic racism [31,32]. We have already initiated a minority-focused (i.e., Black/African American) environmental health education and tracking study that uses qualitative and quantitative data to develop culturally appropriate educational EDC EHL materials and behavior change recommendations. Our goal is to improve EHL and readiness to change, increase exposure-reduction behaviors, and reduce EDC metabolites among marginalized populations. In the current study, despite the challenges of assessing EHL, responses to three survey questions provided support for the effectiveness of the intervention. Participants learned that not all chemicals are bad, and they increased their avoidance of car exhaust and cleaning product fumes. These findings provide encouragement for future research on effective interventions to increase EHL and health protective behaviors.

### 4.3. EDC Exposures Varied by Demographics

Baseline EDC biomarker levels varied by age, sex, and health status. Younger participants had higher PPB levels, which could be due to using more products or eating more packaged food in which PPB is used as a preservative. We will explore this in future research. PPB was recently banned as a food additive by the state of California, which may lead to lower levels in CA and country-wide, as companies typically avoid creating different formulas for different states. Women had higher phthalate levels (MEP and MECPP) and so did men and women who reported themselves to be in better health (MBP and MECPP). Women have been shown to have higher levels of some phthalates, including MEP, using NHANES data [83]. This may be due to women typically using more personal care products than men [84,85,86,87]. Higher phthalate levels among those reporting a better health status may have been driven by the likelihood of phthalates being present in products used by people who engage in health-promoting behaviors. Indeed, people who take supplements tend to report themselves as healthy [88,89,90], and many supplements have been found to contain phthalates [91,92]. Further, many studies have shown toxic chemicals are present in gymnasiums and sports equipment [93,94,95,96,97,98], and the presence of phthalates in products (such as athletic clothing, yoga mats, and flooring) is likely—an important avenue of further research.

### 4.4. EDC Exposure Was Reduced after Report-Back

After the intervention, EDC biomarker levels decreased for all but one metabolite (MECPP), with a statistically reliable reduction in MBP, indicating a reduction in daily exposure, as spot urine samples have been shown to reasonably predict long-term exposures in adults [99]. Attrition may have played a role in the latter, as only 56 participants submitted their second urine test, making our estimates less statistically reliable. In future studies, we will be increasing engagement with participants and providing incentives to motivate more people to return their second test. The lack of a decrease in MECPP may be due to the fact that it is a high-molecular-weight phthalate found in building materials such as flooring, adhesives, and plumbing [100,101]. These exposures may be harder to avoid than, for example, low-molecular-weight phthalates found in personal care products and fragrances [101].

### 4.5. The Million Marker Service and Report-Back Usability Was High

Usability ratings for the program were very high, particularly for urine collection and shipping. The report-back was rated as easy to understand and helpful by the majority of participants. However, there was room to improve, and we have already made several changes to the report format and content. Some of these changes include clearer graphics, improvements to how we report percentiles of exposure compared to NHANES data, and more nuanced actionable feedback. Ratings for the mobile app were less strong. Although optional use of the exposure journal was high, we believe this was one of the main difficulties of using the app. We are currently testing a personalized service to help users document their exposure sources and have developed a new web portal for kit registration, exposure journaling, and report delivery.

### 4.6. Previous Studies of EDCs, EHL Interventions, and Report-Back

Several studies have assessed the importance and efficacy of EDC EHL intervention and/or exposure report-back on increased EHL and exposure reduction. El Ouazzani and colleagues found that EHL education in pregnant women (i.e., three EDC EHL workshops during pregnancy in 268 women) improved risk perception but had no statistically discernible effect on EDC-reduction behavior (i.e., reducing canned food intake and use of paraben-containing products) or reducing BPA or parabens in urine. They concluded that educating women early in pregnancy, taking into account their EDC knowledge, attitudes, and practices, would likely improve outcomes [102,103,104]. These studies, however, did not provide EDC report-back as an intervention for behavior/knowledge modification. In another intervention study, researchers modified EDC exposure behavior by providing a “fresh food” diet, limiting plastic packaged food consumption in participants (*N* = 20). They found reductions in urine levels of BPA and phthalate metabolites after three days on the fresh food diet [25]. While the results indicate that behavior (i.e., diet) changes can reduce EDC exposures, it is unclear whether these reductions could be manifested or maintained in real-world conditions. Our study indicates that EDC EHL education, with personalized recommendations based on report-back, can improve behaviors and reduce exposures in uncontrolled, real-world environments. We are currently testing whether these changes are maintained long-term. Lastly, a study of EDC report-back in women (*N* = 135) randomly assigned half the participants to receive personalized report-back with actionable feedback and the other half to receive study-wide results. They found statistically discernible and/or clinically meaningful changes in behaviors to reduce certain chemicals (i.e., per- and polyfluoroalkyl substances) but not others, as well as slight increases in EHL (which was also high at baseline) [104]. However, this study did not follow-up with EDC exposure testing after report-back.

Biomonitoring studies have been employed for decades to study large-scale toxic exposures. Individual report-back is a newer practice, and along with it comes ethical and logistical challenges. Researchers are concerned with legalities, report reception, and causing undue worry of the participants and communities, especially in vulnerable populations [35,40]. However, several studies have shown that providing report-back with personalized, actionable recommendations has had a positive effect on reducing exposure-causing behaviors in individuals, has been generally well received by communities, has engaged the population in the scientific process, and, in fact, is extremely beneficial for increasing EHL and highlighting environmental justice issues in vulnerable and minority populations [36,37,38,39,40,41,105,106,107,108]. The MM report provides tailored and personalized recommendations for behavior changes and product swaps, using positive, empowering language to encourage behavior changes while minimizing anxiety. Similar to previous research, we also found our report-back service to be well-received by participants, with statistically discernible and/or clinically meaningful changes in EDC exposure and exposure-reduction behavior. Furthermore, future research will focus on adapting the MM service and report-back to empower vulnerable/marginalized populations.

### 4.7. Limitations

There were several limitations to this study, many of which would be improved with further research. A non-randomized, pre–post design like ours can suggest that an observed effect is due to the intervention itself. However, without distinct treatment and control groups, part of the observed effect may be due to factors other than the intervention (like the passage of time or the effect of being enrolled in a scientific study). We are currently conducting a follow-up randomized controlled trial to provide more definitive intervention effect estimates. Although our sample size is comparable to that of other EDC report-back studies [25,102,109], a larger sample would of course further increase the precision (i.e., statistical reliability) of effects. Our sample of participants were all from a limited geographic area (i.e., Nevada), mostly women, White, and with relatively high educational levels and income. Thus, these data are difficult to generalize to a broader, more diverse population. Sex and race/ethnicity play a large role in personal care product choice and cleaning practices, and income can dictate one’s ability to afford healthier products, which are often higher-priced. The lack of specificity and sensitivity in the EHL survey was another limitation of this study. Our EHL survey was adapted from the only validated survey of its kind [29]; however, the survey was not developed specifically for determining EDC-related health literacy and, thus, was not as sensitive for determining EDC-specific EHL, as we would like. The difficulty in assessing low levels of EDC metabolites was also a challenge, as the current EDC testing panel has high detection limits for some of the metabolites (e.g., BPA). However, lowering the detection limits on these metabolites is currently cost-prohibitive for our single-panel test. We experienced a high attrition rate for the urine tests. This was likely due to a long lag time without enough engagement and insufficient incentives. We plan to increase engagement and incentives in future studies. Furthermore, because these individuals were recruited from a larger study, and did not specifically seek out the MM services, it is possible that this population might be less motivated to change. Thus, introducing actionable (and possibly expensive) recommendations could deter rather than motivate them. On the other hand, individuals who are actively seeking to reduce toxic chemical exposures might be more likely to increase their readiness to reduce exposures when given education on how to do so.

### 4.8. Future Directions

Although we found improved readiness to change in women after our intervention, the opposite was seen in men. Thus, in future research, we aim to identify male-specific challenges to exposure reduction, as well as develop more tailored feedback for men. Similarly, we will be adapting, tailoring, and testing our report-back and intervention programs to better engage and support more diverse, vulnerable, and marginalized populations. Additionally, little is known about the EHL status, particularly regarding EDCs, among health professionals, although it is known to be low [110]. Thus, assessing and improving EDC-specific EHL in health professionals, as well as promoting and supporting patient EDC screening and counseling by healthcare providers (through, for example, Continuing Medical Education courses), is another focus of our future work. Currently, we are expanding our sample size for a larger, randomized clinical trial report-back study and are testing a multi-week EHL education curriculum and coaching program, akin to the Diabetes Prevention Program [111], to further promote EHL and EDC exposure-reduction behaviors.

## 5. Conclusions

In this first-of-its kind pilot study, we found that report-back of EDC exposures, along with personalized, actionable feedback, increased exposure-reduction behaviors and reduced EDC exposure in women and men and improved readiness to reduce exposures in women. In men, there was a reduction in readiness to reduce exposures, possibly due to a perceived lack of empowerment or need to change. Future work will focus on identifying the reasons for this sex difference, as well as adapting and improving the MM report-back service to serve a more diverse population. With improved metrics (e.g., an EDC-specific EHL survey) and adaptations to the intervention, we will aim for even greater EDC exposure reductions in both men and women from a broad range of demographic groups.

## Figures and Tables

**Table 1:** Demographic characteristics of participants who completed pre-test surveys (*N* = 434).

Demographics	Mean (Range)	Standard Deviation *
Age	30.7 (18–40)	4.9
BMI	27.1 (17–63)	6.4
Sex	Number	Percentage *
Female	328	75.9
Male	104	24.1
Race/Ethnicity		
Asian	12	2.8
Black	2	0.5
Hispanic or Latino	44	10.1
Native American	3	0.7
Pacific Islander	7	1.6
White	344	79.3
Other	22	5.1
Education		
No high school diploma	4	1.4
GED or equivalent	6	2
High school graduate	18	6.1
Some college but no degree	56	19
Associate degree	47	15.9
Bachelor’s degree	95	32.2
Master’s degree or higher	69	23.4
Household Income		
Less than USD 35,000	49	16.7
USD 35,000 to USD 49,999	56	19
USD 50,000 to USD 74,999	54	18.4
USD 75,000 to USD 99,999	60	20.4
More than USD 100,000	75	25.5
Self-reported health		
Poor	4	1.3
Fair	15	5.1
Good	116	39
Very Good	131	44.1
Excellent	31	10.4

* Valid percentages are shown. A total of 140 participants had missing data for BMI, education, income, and self-reported health; fewer than two people had missing data for all other variables.

**Table 2:** Responses to Environmental Health Literacy Survey Questions (*n* = 434).

	Strongly Disagree	Disagree	Undecided	Agree	Strongly Agree
Chemicals can be found in carpet, rugs, curtains, and furniture	0%	0.90%	4.20%	39.50%	55.40%
Chemicals are always bad for my health *	10.60%	32.10%	22.90%	21.90%	12.50%
I am concerned about the chemicals I am exposed to on a daily basis	1.20%	8.30%	16.60%	45.70%	28.20%
	Never	Hardly Ever	Sometimes	Often	Always
I avoid inhaling car exhaust *	1.20%	2.80%	12.30%	40.40%	43.40%
I avoid inhaling cleaning products *	0.70%	6.00%	23.40%	45.10%	24.80%
I avoid exposing myself and my family members to harmful chemicals	0.20%	1.90%	23.10%	47.70%	27.10%

* Indicates statistically discernible changes after intervention via Wilcoxon signed rank tests.

**Table 3:** Concentrations of biomarker metabolites (ng/mL; *n* = 191).

Metabolites *^a^*	LOD *^b^*	% above LOD	Quartiles (25/50/75)	Specific Gravity Adjusted Quartiles	Creatinine Adjusted Quartiles	NHANES (*n* = 1768) Quartiles
Phthalates						
MBP	0.5	70.7	0.3 48.8 86.2	0.7 46.0 83.6	0.5 31.1 56.5	*^d^* Sum of MBP and MEP:
MEP	0.6	63.4	0.3 33.5 83.6	0.5 33.8 109.0	0.3 22.8 73.6	25.4 45.4 106.1
*^c^* MEHP	0.75	0.5	0.4 0.4 0.4	0.3 0.4 0.5	0.2 0.30 0.3	*^d^* Sum of MEHP, MEHHP, and MECPP:
*^c^* MEHHP	0.5	24.1	0.3 0.3 0.3	0.2 0.3 1.1	0.1 0.2 0.7	9.2 14.1 23.0
MECPP	0.5	88	1.1 2.0 4.3	1.3 2.3 4.1	0.9 1.5 2.8	
Bisphenols						
*^c^* BPA	0.4	1	0.2 0.2 0.2	0.2 0.2 0.3	0.10.10.2	0.6 1.0 1.8
*^c^* BPF	2.5	1	0.3 0.3 0.3	0.2 0.3 0.3	0.10.20.2	0.1 0.3 0.7
*^c^* BPS	0.25	0.5	0.1 0.1 0.1	0.1 0.1 0.2	0.10.10.1	0.3 0.5 1.2
Parabens						
MePB	0.25	64.4	0.1 6.2 31.8	0.2 6.6 32.4	0.14.4 21.9	9.3 43.8 175.9
*^c^* EPB	0.25	17.8	0.1 0.1 0.1	0.1 0.1 0.3	0.10.10.2	0.7 1.4 5.4
PPB	0.25	38.2	0.1 0.1 4.5	0.1 0.2 6.1	0.10.14.1	0.9 5.1 33.5
*^c^* BUP	0.25	2.6	0.1 0.1 0.1	0.1 0.1 0.2	0.10.10.1	0.0 0.1 0.2
*^c^* OBZ	50	12.6	25.0 25.0 25.0	21.2 28.0 43.8	14.3 18.9 29.6	3.8 10.9 44.6

*^a^* Metabolites: MBP = monobutyl phthalate; MEP = monoethyl phthalate; MEHP = mono(EthylHexyl) phthalate; MEHHP = mono-(2-ethyl-5-hydroxyhexyl) phthalate; MECPP = mono-(2-ethyl-5-carboxypentyl) phthalate; BPA = bisphenol A; BPF = bisphenol F; BPS = bisphenol S; MePB = methylparaben; EPB = ethylparaben; PPB = propylparaben; BUP = butylparaben; and OBZ = oxybenzone. *^b^* LOD = level of detection; concentrations below the LOD were replaced by LOD/2. *^c^* These metabolites were removed from further analyses because too few values were above the LOD. *^d^* Sums of low molecular weight phthalates (MBP and MEP) and high molecular weight phthalates (MEHP, MEHHP, and MECPP) were reported in NHANES.

**Table 4:** Demographic variables by metabolite concentration.

	MBP (LMW Phthalate)	MEP (LMW Phthalate)	MECPP (HMW Phthalate)	MePB (Paraben)	PPB
					(Paraben)
Age	*r_s_*(190) = −0.05	*r_s_*(190) = 0.03	*r_s_*(190) = 0.01	*r_s_*(190) = −0.12	***r_s_*(190) = −0.16**
	*p* = 0.52	*p* = 0.68	*p* = 0.87	*p* = 0.11	*p* = 0.03 *
BMI	*r_s_*(134) = −0.14	*r_s_*(134) = −0.02	*r_s_*(134) = 0.03	*r_s_*(134) = −0.12	*r_s_*(134) = −0.06
	*p* = 0.11	*p* = 0.78	*p* = 0.72	*p* = 0.18	*p* = 0.50
Sex	*U* = 3261.50	***U* = 2654.50**	***U* = 2451.50**	*U* = 2982.00	*U* = 3117.00
	*z* = −0.39	***z* = −2.29**	***z* = −2.93**	*z* = −1.26	*z* = −0.84
	*p* = 0.70	*p* = 0.02 *	*p* = 0.003 *	*p* = 0.21	*p* = 0.40
Race/Ethnicity (White vs. non-White)	*U* = 2770.00	*U* = 2432.00	*U* = 2701	*U* = 2797.00	*U* = 2547.00
	*z* = 0.14	*z* = −1.04	*z* = −0.10	*z* = 0.23	*z* = −0.64
	*p* = 0.89	*p* = 0.30	*p* = 0.92	*p* = 0.82	*p* = 0.52
Education	χ^2^(2) = 1.44	χ^2^(2) = 1.50	χ^2^(2) = 0.54	χ^2^(2) = 2.93	χ^2^(2) = 3.07
	*p* = 0.49	*p* = 0.47	*p* = 0.76	*p* = 0.23	*p* = 0.22
Income	χ^2^(2) = 1.24	χ^2^(2) = 1.08	χ^2^(2) = 0.26	χ^2^(2) = 2.21	χ^2^(2) = 3.64
	*p* = 0.54	*p* = 0.58	*p* = 0.88	*p* = 0.33	*p* = 0.16
Health Status	***U* = 2919.00**	*U* = 2142.00	***U* = 2964.00**	*U* = 2537.50	*U* = 2634.50
	***z* = 2.99**	*z* = −0.55	***z* = 3.19**	*z* = 1.25	*z* = 1.70
	*p* = 0.003 *	*p* = 0.59	*p* = 0.001 *	*p* = 0.21	*p* = 0.09

Metabolites: MBP = monobutyl phthalate; MEP = monoethyl phthalate, MECPP = mono-(2-ethyl-5-carboxypentyl) phthalate, MePB = methylparaben, and PPB = propylparaben. LMW = low molecular weight. HMW = high molecular weight. Statistical tests: Mann–Whitney U tests were run for sex, race/ethnicity, and health status; Kruskal–Wallis H tests were run for education and income; Spearman’s rank order correlations were run for age and BMI. * Associations with statistically significant *p* values are shown in **bold**.

**Table 5:** Concentrations of biomarker metabolites pre- and post-intervention (ng/mL; *n* = 48).

Metabolite	Pre-Intervention Mean (sd)	Post-Intervention Mean (sd)	Paired-Samples *t*-Test (One Sided)
MBP (LMW phthalate)	83.9 (108.1)	26.9 (46.7)	t(47) = 3.36, *p* < 0.001
MEP (LMW phthalate)	202.4 (560.1)	127.1 (198.5)	t(47) = 0.94, *p* = 0.177
MECPP (HMW phthalate)	4.8 (15.3)	8.3 (12.2)	t(47) = −1.2, *p* = 0.124
MePB (paraben)	78.0 (201.8)	72.2 (145.2)	t(47) = 0.3, *p* = 0.399
PPB (paraben)	42.0 (121.6)	23.5 (57.5)	t(47) = 1.2, *p* = 0.117

Metabolites: MBP = monobutyl phthalate; MEP = monoethyl phthalate, MECPP = mono-(2-ethyl-5-carboxypentyl) phthalate, MePB = methylparaben, and PPB = propylparaben. LMW = low molecular weight; HMW = high molecular weight.

## Data Availability

Data is available upon author request.

## Abbreviations

EDC	endocrine-disrupting chemical
EHL	environmental health literacy
BP	bisphenol A
BPS	bisphenol S
BPF	bisphenol F
MBP	monobutyl phthalate
MEP	monoethyl phthalate
MEHP	mono(EthylHexyl) phthalate
MEHHP	mono-(2-ethyl-5-hydroxyhexyl) phthalate
MECPP	mono-(2-ethyl-5-carboxypentyl) phthalate
MePB	methylparaben
EPB	ethylparaben
PPB	propylparaben
BUP	butylparaben
OBZ	oxybenzone
